# Everybody's Page

**Published:** 1891-08-22

**Authors:** 


### Everybody's Page.

At first sight the fees in the London Medical Schools would seem to be much higher than those in American colleges, where the average is rather less than half. The extra cost however, in England, is counterbalanced by the fact, that the London schools give nearly twice as long a period of instruction.

Dr. E. H. F. Nuttall, of John Hopkins University, is evidently a man of cheery disposition. He encourages us with the assertion, that phthisical patients expectorate from 250,000 to 4,000,000,000 bacilli in twenty-four hours! We ought to be grateful to him and all the ancestral Nuttalls for spending their lives in counting up these figures, for the family must have been busy for some generations past.

Experience, or rather abstinence, has not taught us how much truth there is in the assertion that tea-drinking is a cause of cold feet. The suffering caused, at night more especially, by this distressing infliction is sufficient to make any test or sacrifice worth while. Those who suffer from cold feet, and are habitual drinkers of tea at breakfast and in the afternoon, may be able to confer not only a boon upon themselves but upon their fellow-sufferers by making an experiment in this direction.

Anyone who suspects the presence of arsenic in their wall-paper can put the accuracy of their suspicion to the test in the following simple manner: Dip a small piece of the paper in strong ammonia water. If arsenic is present a bluish colour will appear. In order to make certain doubly sure, a crystal of nitrate of silver can be moistened with a drop of this fluid. This further test will show if the colour is due to arsenic, as, if it is, a deposit of a yellow tint will be formed on the crystal.

The greatest obstruction in the way of the accomplishment of Dr. J. Roberts Thomson's hope, which is that of all educated people, that in a few years zymotic diseases will be reduced to very small proportions by means of improved organization and increased scientific control, is the apathy which exists in country districts and small towns over sanitary work. This want of interest is almost universal, and is not the only difficulty to be contended with. The medical officer of health cannot be too highly trained, and above all things he should be absolutely independent of all local jealousies, and, as Dr. Thomson suggests, freed from such restraints as the present uncertain tenure of his office inevitably causes.

Such general interest is now being taken in the question of a scientific disposal of our dead that the time cannot be far distant when our present very unscientific method of burial is abolished. Early next year a strongly-signed memorial is to be presented to the Prime Minister asking for concentration in some central authority of the control now divided between the Home Office and the Local Government Board, and a further step is to be taken in the shape of a memorial to the County Council to take into consideration the question of sanitary burial. Here is an opportunity for the Council to distinguish itself, by doing some good for the public it is meant to serve instead of airing useless and frequently harmful individual crochets.

It has often been complained, that while many men in this country who have prospered in commerce have been elevated to the peerage, this honour has not been conferred on any eminent medical men. Whatever justice there is in this complaint in England, there is no longer ground for it in Japan, where the Emperor has nominated Dr. Hide Miyake, Professor in the Medical Faculty of the Imperial University of Tokio, a member of the House of Peers.

At a recent meeting of the Paris "Society of Hypnology" Dr. Bernheim combatted very energetically the opinion of those who maintain that hypnotism is a nervous condition analogous to or identical with hysteria. The hypnotic sleep is not the equivalent of a hysterical crisis, he considers, and the fact that a person can be hyptonised is no proof that he or she is hysteric; in fact, hypnotism is a normal condition which is met with amongst all subjects, and which has nothing in common with hysteria.

An Italian authority makes some sensible, if not altogether novel, remarks on the subject of protection against cold. Many people are prone to put too much faith in the beneficial effects of a fire, forgetful of the fact that a fire fails to warm the body equally. It also acts principally on the surface of the body, and is apt to do more harm than good. Movement and exercise, on the other hand, promote the warmth of the body, increase the circulation and breathing power, and strengthen the appetite. These, in the case of full-blooded people, combined with strengthening food and tonics, will be found to prove most advantageous in overcoming cold.

Though the intelligence of the savage does not rank high, his ingenuity often argues that his intelligence is latent rather than absent. The inhabitants of the island of Isabelle, one of the group of the Solomon islands, live in remarkable houses perched high up in large trees. The approach to these houses is by a ladder, which is drawn up at night or on the appearance of an enemy. The latter not only has no means of getting to close quarters with the party he wishes to attack, but has a somewhat uncomfortable quarter of an hour, if he is foolish enough to stop so long under the tree, as the Isabellan keeps a large store of heavy stones in his house which are apt to discompose the headgear of his assailants.

The result of the establishment of free libraries in Paris has been very similar to that in this country, and points to the fact that human nature is much the same all the world over. Out of 1,386,642 books given out to read at the various libraries last year half were novels. The French artisan, like the British, prefers works of fiction to those of a more solid and instructive kind. Forgetful of the fact that you may lead a horse to water but cannot make him drink, the administration instructed the librarians to bring their influence to bear upon the frequenters of their libraries and get them to read more instructive literature. Like good citizens they did as they were told, but novel reading did not cease, whereas the number of readers decreased in a very remarkable manner. The instructions to the librarians have now been withdrawn.

